# Medical educators’ perception of communication training with simulated patients: an explorative study approach

**DOI:** 10.1186/s13104-017-2988-8

**Published:** 2017-11-29

**Authors:** Simone Alvarez, Jobst-Hendrik Schultz

**Affiliations:** Department of General Internal Medicine and Psychosomatics, University Hospital Heidelberg, Im Neuenheimer Feld 410, 69120 Heidelberg, Germany

**Keywords:** Simulated patient, Simulation training, Communication, Educator perception, Medical education

## Abstract

**Objective:**

Medical students’ perceptions of the use of simulated patients (SP) in communication training in medicine have been studied intensively, but insights about faculty perception of this type of simulation training remain rare. This study aimed to assess medical educators’ perception of the relevancy of SP communication training, as well as its closeness to reality. Medical educators were surveyed by standardized questionnaire and open-ended questions. The questionnaire allowed educators to rate several aspects of the training such as its closeness to reality and relevancy to real-life physician–patient interaction.

**Results:**

Educators’ perception of relevance and realism of SP training increases with teaching experience. This appears to be influenced by factors such as internal or external status of the educator, personal experience with communication training during medical studies, as well as medical field taught. Communication training with SP is valued highly by medical educators mainly because of its versatility and broad spectrum of applicability. The wide range of application of SP in medical education seems most evident to senior educators because of their increased amount of experience with physician–patient interaction, whereas junior educators appear often hindered by the aspect of simulation caused by the thought that the patients are “merely” actors.

## Introduction

Reviews of communication skills training in medical education have provided evidence that interactive teaching methods are among the most successful [1, 2]. A truly interactive teaching and learning approach is the one of simulated patients (SP). A SP is a person who is trained to realistically portray a patient with a certain medical condition so that medical students may practice teaching communication, but also clinical or physical examination skills in a safe and controlled environment that provides opportunity for learning through feedback. Training with SP allows for practice until an adequate skill level, or feeling of confidence is reached by the student [3]. This aspect becomes especially important in emotionally challenging situations such as during delivery of a terminal diagnosis [4]. SP can be trained in number of clinical cases, thus providing a broad range of application, and in a way that matches the student’s current level of achievement, which leaves the educator with maximum control of the decision about how a given learning goal, which is usually pre-defined as a specific skill, can be achieved [5]. At a large German medical faculty, SP are used as a regular part of the medical curriculum during instruction in the following five subjects/disciplines in the field of internal medicine: hematology, gastroenterology, pulmonology, geriatrics, and psychosomatics. As illustrated in Table 1, each subject has its own pre-determined learning objectives which the medical educators have to take into consideration when teaching.

**Table 1:** Communicative content by subject

Subject	Content
Hematology	Curative and palliative consultation
Gastroenterology	Medical history taking, communication of findings
Pulmonology	Emergency medical history taking, patient delivery
Geriatrics	Medical history taking, communicating with relatives
Psychosomatics	Confrontation, criticism

Usually, SP training is regarded as very valuable by students [i.e. 6–9]. However, insights about educator perception of this type of simulation training remain rare. Consequently, this study aimed to explore educators’ opinion about SP communication training, and also their perception of its relevancy and its closeness to reality.

## Main text

### Methods

During two consecutive semesters, all 44 medical educators who taught classes during the clinical block of internal medicine during the academic year of 2015 were surveyed by standardized questionnaire after having taught SP training sessions. Therefore, no sampling was necessary. The questionnaire allowed the participants to rate several aspects of the training such as its closeness to reality and relevancy to real-life physician–patient interaction on a set scale. Open questions provided further opportunity to reflect the training.

#### Sample and data collection

All 44 medical educators whom have taught communication classes with SP during the course of the two semesters were asked by a member of the research team to participate in the study. 38 (86%) medical educators completed and returned the questionnaires immediately after having taught the class.

#### Data analysis

All quantitative data were calculated using SPSS 22. Open questions were analyzed according to qualitative content analysis according to Mayring using an inductive approach where specific statements are systematically coded and clustered in summarizing categories. In order to ensure accuracy of the process, the coding was done by two researchers simultaneously and later on compared [10].

### Results

#### Quantitative results

Since the data did not meet the assumptions for parametric testing (small sample size, unequal group size, non-normal distribution), and the non-parametric Mann–Whitney-U statistic independent samples test was chosen for data analysis. Given the unequal distribution of the data, the median seemed the appropriate statistic for interpretation. To determine statistical significance, the confidence level was set at 95%. Descriptive statistics of medical educators can be found in Table 2.

**Table 2:** Medical educator descriptives

	Total faculty *N*	Junior faculty (≤ 5 years) *n*	Senior faculty (≥ 6 years) *n*
University status			
Internal	19	12	7
External	19	2	17
Communication training during medical studies			
Did attend	15	7	8
Did not attend	23	6	17

N, number

As Table 3 shows, educators’ perception of relevance and realism of SP training appears to increase with teaching experience. Educators with 5 years or less of overall teaching experience rated communication training with SP less relevant *U*(14,24) = 93, *p* = .022 than educators with 5 or more years of overall teaching experience at the higher education level and also as less realistic than those with 5 or more years of overall teaching experience at the same level. At the medical faculty where the study took place, educators who teach SP training can either have internal status, meaning that they are regularly employed by the same university they teach SP training sessions at, or external status, meaning that they are not directly employed by the university, but have a teaching obligation. Table 3 also illustrated that when comparing educator opinion according to status, SP training was rated much more positively by external educators, meaning by those who are working either in private practice or are employed by hospitals elsewhere. Analysis showed that external educators found the SP training more relevant *U*(19,18) = 103, *p* = .039 and also more realistic than educators with internal status. When comparing educator perception based on the fact if they had attended some sort of communication training themselves during their medical studies, it was found that those who did not attend, rated the training to be more relevant *U*(15,20) = 101, *p* = .036 and also as more realistic than those who did attend. Three educators did not indicate whether they had received communication training during medical studies.

**Table 3:** Results medical educators

	Relevancy	Realism	*n*
	*Mdn*	*Mdn*	
Educators ≥ 5 years of teaching experience	8.5*	8.5	24
Educators ≤ 6 years of teaching experience	7.5*	6.6	14
External educators	8.8*	8.1	18
Internal educators	7.8*	7.1	19
Educators with CT	7.6*	6.7	15
Educators without CT	9.1*	7.9	20

Scale: 1 = not very relevant/realistic to 10 = very relevant/very realistic

CT, communication training

* Statistically significant p < .05

Table 4 illustrates a comparison by type of medical field taught by utilizing SP. Here, relevance was rated highest by educators who taught communication in geriatrics followed by hematology and gastroenterology. A comparison of realism of SP communication training yields somewhat different results. Educators teaching gastroenterology rated SP training as highly realistic, followed by educators in geriatrics and pulmonology, whereas those who taught hematology and psychosomatics found SP training to be less realistic.

**Table 4:** Results based on medical specialty

	Relevancy	Realism
	*Mdn*	*Mdn*
Geriatrics	8.8	7.5
Hematology	8.7	6.7
Gastroenterology	8.5	8.0
Pulmonology	7.6	7.4
Psychosomatics	7.2	6.4

Scale: 1 = not very relevant/realistic to 10 = very relevant/very realistic

#### Qualitative results

Educators were asked about the aspects of SP communication training they perceived as positive or as in need of improvement. On the positive spectrum, many of the educators named SP training as a valuable opportunity for students to self-reflect, mainly through video analysis, through SP feedback, through peer feedback, and through educator feedback. Many also named the quality of SP performance and realistic scenarios as positive aspects of SP training. Interestingly, mainly experienced educators with more than 6 years of teaching experience described SP training as an experiential and interactive teaching method, which is valued because it allows for a certain degree of freedom in teaching, and occasionally even provides educators with an opportunity to learn something new themselves. On the negative spectrum, educators indicated that students are often lacking the necessary medical knowledge needed to manage the lessons effectively. Or that the time slots provided for SP training are too short to properly conduct and reflect each conversation. Also, some educators noted unmotivated and unprepared students as a negative aspect during SP training sessions. Few named inadequate quality of SP performance and training scenarios as negative aspects of SP training.

### Discussion

The great lack of literature and publicly available information on the subject of faculty training for communication sessions with SP as well as on the faculty opinion of the training, makes it very difficult to tie the results of the findings to existing literature. However, the present results indicate that experienced educators recognize the many possibilities and options of application SP training provides more often than those with less experience. Possible reasons for this could be their increased amount of experience with real patients, and their broader range of real-world experiences. Another reason could be that teaching confidence increases with teaching experience. More confident educators may be more inclined to make use of the opportunity to experiment with SP as instructional tools, and feel more comfortable taking on a less active role during the teaching process [11]. However, another way to gain confidence is through thorough preparatory training during which the potential of simulation training could be made subject [12]. The similarities between the results of experienced/inexperienced and internal/external educators can be attributed to the external educators being mostly of the experienced category. Only two external educators in our sample had 5 years or less of teaching experience.

During this study, perceptions of educators who have had some sort of communication training during their own medical studies with the ones who did not were compared. Surprisingly, six educators of the inexperienced category indicated that they had not attended communication training during their medical studies, whereas 17 of the experienced category reported that they had not. The question why those who did attend communication training during their own medical studies rate SP training significantly less relevant and also less realistic will be explored further by a qualitative follow-up study.

The qualitative results suggest that the difference in perception of communication training with SP seem to be influenced strongly by the pre-set content of the individual training as well as the portrayed pre-set scenario. At this particular faculty, scenario cases are written by medical professionals of the various specialties, and are usually approved by an independent expert of the particular field before an SP is trained on the role, and the case is used in medical student training [13]. In particular, it was found that educators’ opinion of the closeness of reality of the communication training in psychosomatics is slightly lower than the rating of the other fields. The reason for this could be that specific scenarios are more difficult for students to handle than others. For example: during the first part of the psychosomatic training students have to confront a patient with anorexia about not abiding by the previously agreed upon eating contract. Naturally, a conversation that is confrontational in nature is received differently depending on a student’s prior experiences with this type of situation, and is perceived difficult even for experienced physicians [14]. Consequently, the portrayed scenario is judged in accordance with individual perception. Another medical specialty that differed from the others was hematology. Just as psychosomatics, this training session was rated as highly relevant but not as particularly realistic. A reason for this could be that the scenarios during this training are also challenging: students have to inform a patient about the specifics of his or her illness: In one case the illness can be cured, in the other case the patient is terminally ill. Specifically the latter is often experienced as difficult and emotionally difficult by medical students [15]. This illustrates the importance of the quality of the scenario and leads to the assumption that some scenarios have to be frequently re-evaluated by experts of the field, and checked again for closeness to reality. It also illustrates the importance of a frequent quality check of SP performance for accuracy. The overall lower rating of closeness to reality by faculty of all medical fields may stem from the fact that even though SP are rated by educators as versatile tools of medical education, they are after all, still perceived as actors. The qualitative results underline this assumption. In this study, some medical educators explicitly named quality of SP performance as a positive factor, but in some cases, the same was explicitly named as a negative aspect and a hindrance to training success and student motivation. Overall, the findings of this study highlight the importance of thorough faculty training in preparation of SP training sessions. During regular faculty training, the value and potential of SP training should be illustrated and important information about how to successfully handle aspects such as time management and student motivation should be given.

## Limitations

A limitation of this study was the somewhat small number of participants due to the fact that only faculty from the field of internal medicine of a single institution were surveyed. In quantitative terms, a larger number of faculty members from various disciplines would have provided for a more comprehensive view of educators’ perception of SP training. However, the knowledge derived provides important ground work for further hypothesis development. Additional research should be conducted between the relationship of teaching experience and perception of communication training with SP.

### Conclusion

All over the globe, simulated patients are successfully used in the training of medical professionals and much care is taken to ensure that these training sessions are as effective as possible. However, the results of this study suggest that the same care should be paid to faculty training in preparation of communication training sessions with simulation patients. Proper training should prepare educators new to teaching with SP with a skill set necessary to make thorough use of the full potential simulation provides as a teaching method.

## Abbreviation

SP: simulated patient
